# Tropical anurans mature early and die young: Evidence from eight Afromontane *Hyperolius* species and a meta-analysis

**DOI:** 10.1371/journal.pone.0171666

**Published:** 2017-02-09

**Authors:** Ulrich Sinsch, Jonas Maximilian Dehling

**Affiliations:** Department of Biology, Zoology Group, University of Koblenz-Landau, Koblenz, Germany; Universitat Trier, GERMANY

## Abstract

Age- and size-related life-history traits of anuran amphibians are thought to vary systematically with latitude and altitude. Because the available data base is strongly biased towards temperate-zone species, we provide new estimates on eight afrotropical Reed Frog species. A meta-analysis of the demographic traits in 44 tropical anuran species aims to test for the predicted clinal variation and to contrast results with variation detected in temperate-zone species. The small-sized reed frogs reach sexual maturity during the first or second year of life, but longevity does not exceed three to four years. Latitudinal effects on demographic life-history traits are not detectable in tropical anurans, and altitudinal effects are limited to a slight size reduction at higher elevations. Common features of anuran life-history in the tropics are early sexual maturation at small size and low longevity resulting in low lifetime fecundity. This pattern contrasts with that found in temperate-zone anurans which mature later at larger size and grow considerably older yielding greater lifetime fecundity than in the tropics. Latitudinal and altitudinal contraction of the yearly activity period shape the evolution of life-history traits in the temperate region, while trait variation in the tropics seems to be driven by distinct, not yet identified selective forces.

## Introduction

Demographic life-history traits of amphibians are thought to vary systematically with latitude and altitude among species and also among conspecific populations [1]. Available evidence on interspecific variation suggests that in fact average age and longevity augments from equatorial regions towards the poles and also or exclusively with increasing altitude [2–4]. At the intraspecific level, populations of the European anurans *Epidalea (Bufo) calamita* and *Rana temporaria* showed similar trends in the latitudinal and altitudinal variation of age at maturity and longevity, whereas age-adjusted size was insensitive to altitudinal effects and weekly affected by latitude [4–6]. Unfortunately, current evidence available for the analysis of demographic trends is considerably biased towards temperate-zone species (>23.44°N or S) rendering inferences on tropical amphibians inhabiting the equatorial belt from 23.44°N to 23.44°S tentative [7]. Detectable latitudinal variation of traits in tropical species is not probable because the location of the intertropical convergence zone varies over time in annual cycles and moves southwards during the past 600 years [8].

The major source of information on the age of amphibians without previous recapture history is the retrospective estimation by skeletochronology [4, 9, 10]. Lines of arrested growth (LAG) interrupting round bone growth are the result of a genetically based, circannual rhythm synchronised with seasonal cycles, usually hibernation in temperate-zone amphibians [11]. LAGs are also formed in tropical habitats in which seasonality is mainly based on precipitation regime [12]. Skeletochronological studies on the demography of tropical anurans focus currently on Asia [13–15], South America/Caribbean [7, 16], and Madagascar [17, 18], whereas knowledge on afrotropical species is limited to currently three species (S1 Table) [19].

We aim to scale down the apparent gap of knowledge on tropical amphibians and specifically on African frog species by providing demographic data on eight of the eleven currently known *Hyperolius* species (Anura, Hyperoliidae) inhabiting Rwanda [20]. The genus *Hyperolius* is among the most diverse sub-Saharian anuran genera (141 species) [21], but to our best knowledge this is the first report of estimates on demographic key-traits such as age at maturity, longevity and age-adjusted size. Moreover, the populations sampled at latitudes of 1.6–2.6°S are closer to the equator than those of any other anuran species studied skeletochronologically so far, and cover an altitudinal range of 1,643–2,379m asl. Specific aims of our demographic analysis of Afromontane frog species are (1) to describe the post-metamorphic life history represented by age at maturity, median age and longevity, and (2) to identify sex-specific differences in traits and growth patterns in those species represented by larger samples. We complement the new evidence on eight afromontane species with a meta-analysis of published evidence on a total of 37 anuran species inhabiting the tropical belt all over the world (S1 Table). We selected those studies reporting age and size data derived from at least five individuals per gender to test for among-species variation of age and snout-vent length at maturity, and of longevity and maximum size, and their association with latitude and altitude as proxies for environmental variation in the tropics. The first comprehensive and quantitative analysis of demographic traits emphasizes that trait evolution in tropical anurans varies in several aspects from that in temperate-zone anurans.

## Materials and methods

### Study area, species and sampling sites

A total of 267 specimens pertaining to eight *Hyperolius* species were collected during eight field trips (March and October 2009, September-October 2010, March 2011, March-April 2012, March 2013, May 2014, October 2015) taxonomically identified using molecular, bioacoustic and morphological features [22–24]. *H*. *castaneus* (42 males, 15 females, 10 juveniles) originated from an altitudinal transect (1,813m asl at 2.4478°S, 29.1072°E to 2,379m asl at 2.5287°S, 29.3540°E) across the Nyungwe National Park and neighbouring localities in southwestern Rwanda. Nine specimens were kept under controlled conditions (20±2°C, natural LD, moist soil without rainfall simulation) at the University of Koblenz to monitor bone growth and LAG formation during 1.5 years in captivity. *H*. *discodactylus* (5 males, 1 female) originated from the Uwasenkoko swamp (2.379m asl at 2.5287°S, 29.3540°E) in the Nyungwe National Park. *H*. cf. *cinnamomeoventris* (12 males), *H*. *kivuensis* (53 males, 2 females), *H*. *lateralis* (44 males), *H*. *rwandae* (7 males, 2 females) and *H*. *viridiflavus* (47 males, 1 female) were collected in a partially flooded agricultural area (“Marais”) at Huye (1,643m asl at 2.607°S, 29.757°E). Finally, *H*. *glandicolor* (11 males, 15 females) originated from an inselberg plateau (2,287m asl at 1.6438°S, 29.397°E). The strongly male-biased sex ratio was due to the fact that specimens were usually collected by hand during night by tracing advertising individuals.

**Fig 1:**
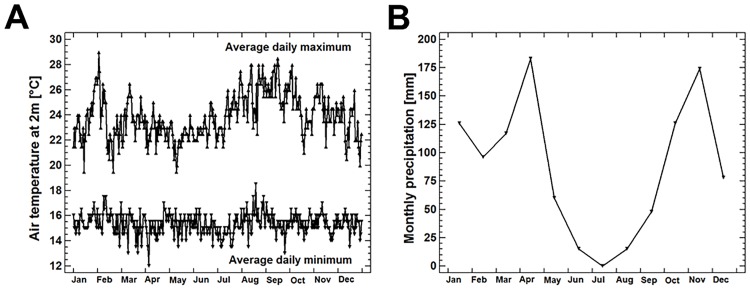
Seasonal variation of temperature (A) and precipitation (B) in Butare, Rwanda. Temperature is presented as average daily minimum and maximum during 2009–2011. Precipitation is presented as the average monthly sum during 2000–2012.

Seasonal variation of air temperature (at 2m above ground in the shade, Fig 1) in Butare was recorded March 9^th^, 2009 to October 26^th^, 2011 using a Tinytag Datalogger (Gemini Data Loggers UK Ltd.). Average monthly variation of precipitation was recorded in the period 2000–2012 and obtained from World Weather online (accessed 15.10.2015).

### Bone sampling and skeletochronological processing

Each individual was sexed and snout-vent length (SVL, distance between snout tip and cloaca) measured to the nearest 0.1mm using a calliper. With the exception of nine *H*. *castaneus* (kept in captivity) all specimens were sacrificed immediately after collection in accordance to the accepted standards of veterinary medicine by exposure to an overdose (buffered 1% solution for five minutes) of the anaesthetic MS-222 (deep anaesthesia within 10–25 seconds). Collection, sacrifice and export of the specimens were approved by the Rwandan Development Board (RDB, national agency of nature conservation). Specimens were stored individually in 70% ethanol at room temperature for future molecular and morphological examination. For skeletochronological age determination the 3^rd^ or 4^th^ digit of a forelimb was toe-clipped, and in some individuals a humerus or femur as well. Laboratory protocols followed the standard methods of skeletochronology [4, 25]. The samples were embedded in Historesin^™^ (JUNG) and stained with 0.5% cresylviolet [26]. Diaphysis was cross-sectioned at 12μm using a JUNG RM2055 rotation microtome. Cross sections were examined light microscopically for the presence of growth marks at magnifications of 400x using an OLYMPUS BX 50. We distinguished strongly stained lines of arrested growth (LAGs) in the periosteal bone, separated by faintly stained broad growth zones, and the line of metamorphosis (LM), separating larval from post-metamorphic bone [4, 27]. We selected diaphysis sections in which the size of the medullar cavity was at its minimum and that of periosteal bone at its maximum. The number of LAGs was assessed independently by the authors to estimate age.

### Meta-analysis of demographic life-history traits of tropical anurans

The tropics are geographically limited to the region between the northern and southern latitude of 23.44°. We considered all published skeletochronological studies on anuran species inhabiting this equatorial belt and reporting the age of at least five individuals per gender. Including our own data we obtained data on a total of 44 species, specifically on males of 43 species and on females of 29 species (S1 Table). If the studies did not explicitly associate age data with gender, we assumed that the majority of data were obtained from males due to the general capture bias towards males (see our *Hyperolius* data as an example). Plasticity of male and female life history was analysed in four traits: (1) minimum age at maturity (n LAGs) = age of the youngest adult of the sample; (2) minimum SVL (mm) of adults = size of the smallest adult irrespective of age; (3) longevity = maximum age detected within a sample (n LAGs); (4) maximum SVL (mm) of adults sampled irrespective of age. As a proxy for the climate at the sampling localities we used latitude and altitude above sea level. If these environmental variables were not explicitly given in the corresponding skeletochronological study, we estimated latitude and altitude by locating the sampling sites in electronic topographical maps. If anuran sampling occurred along an altitudinal transect, we calculated the average altitude to represent the transect climate.

### Statistical analysis

All variables were first tested for normality. As size and age distributions of *Hyperolius* spp. were significantly skewed, descriptive statistics included median, minimum and maximum. Statistical comparison between gender and between taxa was based on the non-parametric Mann-Whitney-Wilcoxon W-test. Growth following metamorphosis was estimated using the von Bertalanffy equation [28]:

$$SVL_{t}=\;SVL_{max}-\;\left(SVL_{max}–\;SVL_{met}\right)\;*\;e^{-k*t}$$

where SVL_t_ = average body length at age t; SVL_max_ = asymptotic body length; SVL_met_ = body length at metamorphosis; t = number of growing seasons experienced (n LAGs), and k = growth coefficient (i.e. shape of the growth curve). SVL_met_ was assessed for each species from tadpoles of Gosner stages 40–43 collected in the field. The von Bertalanffy growth model was fitted to the average growth curve using the least square procedure (nonlinear regression). Estimates of SVL_max_ and k are given with the corresponding 95% confidence interval. Sexual size dimorphism was tested for based on SVL_max_. The absence of overlap between confidence intervals was considered as a significant deviation at P<0.05.

The meta-analysis of size- and age-related life-history traits was performed separately on males and females because pronounced sexual dimorphism is present in many tropical anurans [7, 18]. The association among life-history traits and latitude and altitude was tested for applying a factor analysis. Variables were standardized by dividing the difference between value and arithmetic mean by the standard deviation. Extraction criterion for principal components was an eigenvalue >1. Extracted principal components were submitted an orthogonal VARIMAX-rotation to yield factor loading of original variables close to 1 (strong association) or 0 (no association). Identified associations between a life-history trait and latitude and/or altitude were modelled using regression analyses (selection criterion: maximum R^2^). Significance level was set at alpha = 0.05. All calculations were performed using the procedures of the program package STATGRAPHICS Centurion, version XVI (STATPOINT Inc.).

## Results

### Histological features of round bone diaphysis sections in *Hyperolius* spp.

**Fig 2:**
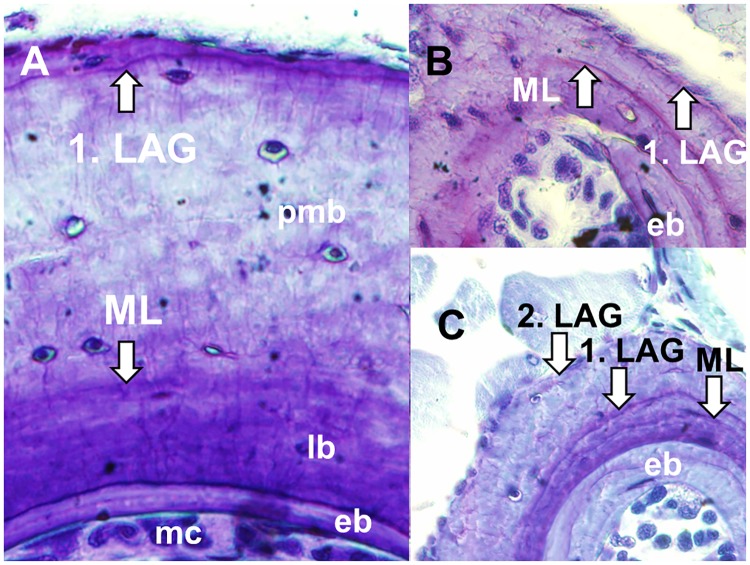
Growth marks in cross sections of *H*. *castaneus* round bones. (A) Humerus (male, 25.9mm SVL); (B) First phalanx of the same male; (C) Second phalanx (male, 24.0mm SVL). Abbreviations: ML = line of metamorphosis, LAG = line of arrested growth, mc = medullar cavity, eb = endosteal bone, lb = larval bone, pmb = postmetamorphic bone.

In all species examined, discernible growth marks were present in the stained diaphysis cross sections of humeri, femura, first and second phalanges (e.g. *H*. *castaneus*, Fig 2). The periosteal bone produced during the larval period stained darker than that produced during the terrestrial stage and was separated by a faint line of metamorphosis (LM). Partial or complete endosteal resorption of LM was observed in all bone types, but varied among species: 0% in *H*. *castaneus*, *H*. *discodactylus* and *H*. *lateralis*, 8.3% in *H*. *glandicolor*, 29.2% in *H*. *viridiflavus*, 60% in *H*. *kivuensis*, 77.8% in *H*. *rwandae*, and 83.3% in *H*. cf. *cinnamomeoventris*. Lines of arrested growth (LAGs) were easily distinguishable from the less-stained growth zones in the post-metamorphic periosteal bone (Fig 2). The number of LAGs was the same in the phalanx and femur or humerus of randomly chosen preserved individuals (two per species) demonstrating that non-lethal phalange sampling allows for precise LAG estimation.

The position of the last LAG and the corresponding collection date of the individual allowed for an estimation of the season in which bone growth was arrested. The periphery of bones collected between March and May showed narrow to broad growth zones without a terminal LAG, whereas the bones of individuals collected in September or October showed a peripheral LAG without or with a very small terminal growth zone. We conclude that growth was arrested during the dry season between June and September (Fig 1). This growth pattern was evident in all species examined. As double lines, i.e. multiple LAGs, were never observed, there was no indication for more than one period of arrested growth per year.

**Fig 3:**
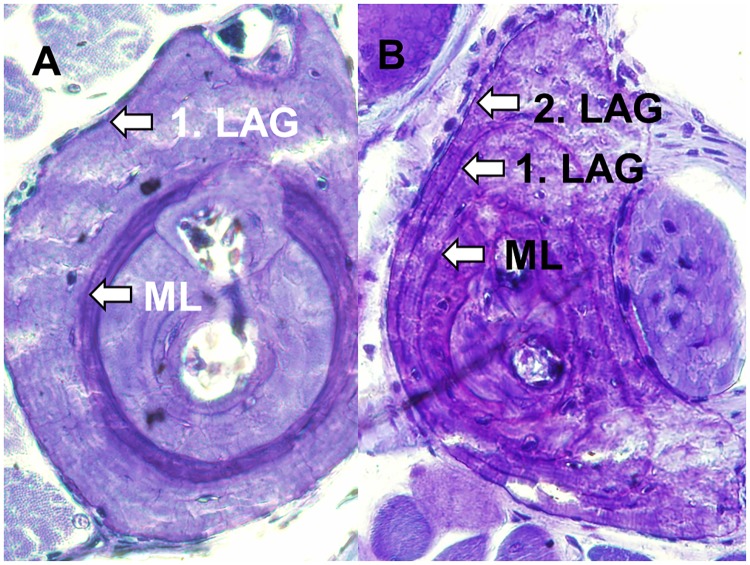
Growth marks in phalanx cross sections of *H*. *castaneus* phalanges kept in captivity. **A** and **B** refer to a male of 26mm SVL, sampled in October 2010 and in March 2012, respectively.

LAG formation was also observed in 7 out of 9 *H*. *castaneus* which were captured in October 2010 and kept in captivity in terraria until March 2012 (Fig 3A and 3B). The LAG formed in captivity was located at the periphery of the bone, with very little additional bone growth. Two individuals did not show any periosteal bone growth during captivity.

### Age distribution and longevity in *Hyperolius* spp

**Fig 4:**
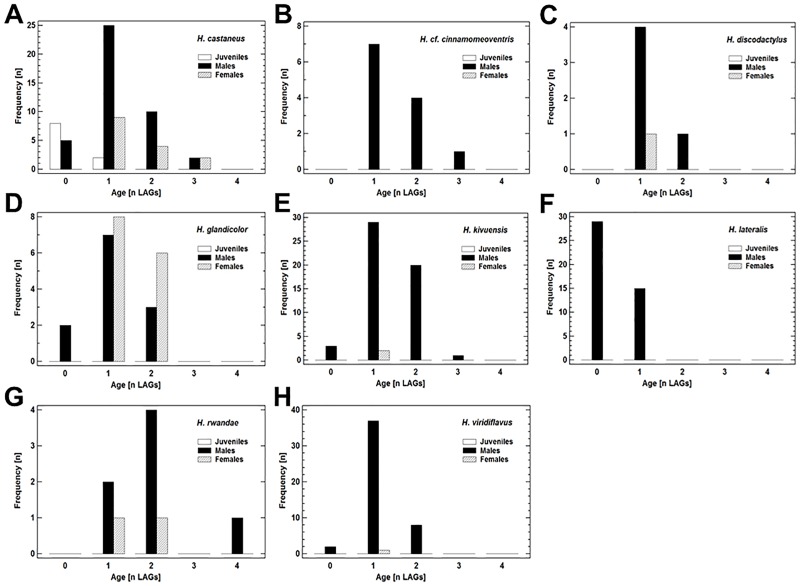
Age distribution of eight *Hyperolius* species. For further details see Table 1 and text.

**Fig 5:**
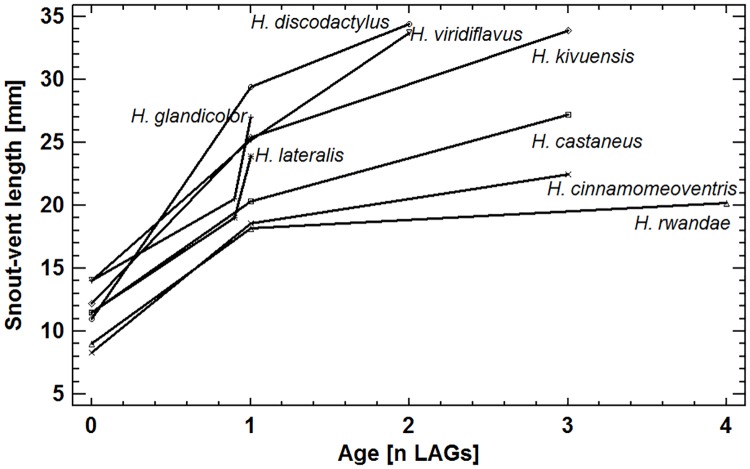
Age-size related life-history features: median SVL at metamorphosis, SVL at sexual maturation and SVL at maximum age.

The *Hyperolius* species studied were generally short-lived with a longevity ranging from one year (= survived dry season) in *H*. *lateralis* to four in *H*. *rwandae* (Table 1, Fig 4). In the samples including a significant number of females, median age did not differ significantly between sexes (*H*. *castaneus*: Mann-Whitney-Wilcoxon W-test, W = 379.5, P = 0.187; *H*. *glandicolor*: Mann-Whitney-Wilcoxon W-test, W = 74.0, P = 0.179; Table 1). Sexual maturation often occurred in the same year of metamorphosis, but at latest following the first dry season (Table 1). Males were considered mature reproductive, if their throats were coloured yellow, females were identified by having egg masses visible through the transparent parts of the abdominal ventral skin. Small-sized species had a greater life expectancy than the large ones (Fig 5).

**Table 1:** Sex- and species-specific features of demographic life-history traits in *Hyperolius* spp. Data deficiency is indicated by? Size at metamorphosis is given as mean, range and sample size.

Species	sex	Size at metamorphosis (mm)	Age at maturity (n LAGs)	Minimum size at maturity (mm)	Median age (n LAGs)	Median size (mm)	Longevity (n LAGs)	Maximum size (mm)
*H*. *castaneus*	♂	11.5 10.0–13.9 n = 5	0	20.3	1 (n = 42)	23.9	3	27.2
	♀		1	24.1	1 (n = 15)	29.0	3	33.0
*H*. cf. *cinnamomeoventris*	♂	8.3 8.0–10.0 n = 3	1	18.6	1.5 (n = 12)	21.4	3	22.5
*H*. *discodactylus*	♂	11.0 11–11 n = 2	1	29.4	1 (n = 5)	32.1	2	34.4
	♀		1	?	1 (n = 1)	40.0	?	40.0
*H*. *kivuensis*	♂	12.2 11.0–16.0 n = 10	0	25.4	1 (n = 53)	27.3	3	33.9
	♀		1	?	1 (n = 2)	34.7	?	36.1
*H*. *glandicolor*	♂	?	0	20.5	1 (n = 12)	23.5	2	27.0
	♀		1	26.0	1 (n = 14)	32.0	2	34.9
*H*. *lateralis*	♂	11.5 10.0–13.0 n = 2	0	19.0	0 (n = 44)	20.4	1	23.9
*H*. *rwandae*	♂	9.0 9.0 n = 1	1	18.2	2 (n = 7)	19.3	4	20.2
	♀		1	23.4	1.5 (n = 2)	23.5	2	23.5
*H*. *viridiflavus*	♂	14.0 13.0–15.0 n = 8	1	25.2	1(n = 47)	26.6	2	33.7
	♀		1	?	1(n = 1)	28.4	?	28.4

### Growth pattern in *Hyperolius* spp

**Fig 6:**
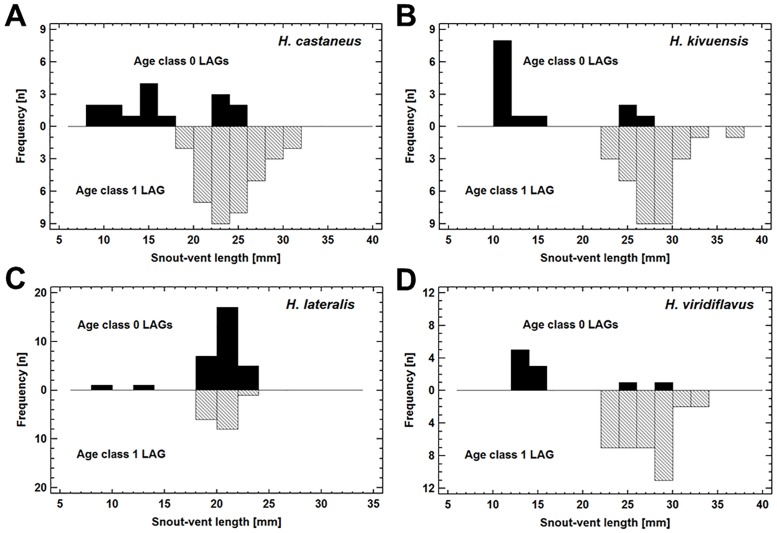
Size distribution of all 0 LAG and 1 LAG individuals of *H*. *castaneus* (A), *H*. *kivuensis* (B), *H*. *lateralis* (C) and *H*. *viridiflavus* (D). All 1 LAG and few 0 LAG individuals were sexually mature (usually males, see Table 1). The bars represent 2mm size classes of the individuals.

Using the von Bertalanffy growth model on the age-size data of amphibians requires knowledge of the snout-vent length at metamorphosis and of the duration of the growth period between metamorphosis and the first arrestment of growth. Size at metamorphosis was available for seven of the eight species and ranged from 8.3mm in *H*. cf. *cinnamomeoventris* to 14.0mm in *H*. *viridiflavus* (Table 1). Metamorphs and tadpoles of all stages were found at the beginning and end of the rainy period suggesting continuous reproductive activity and subsequently continuous recruitment of metamorphs in *H*. *castaneus*, *H*. *discodactylus*, *H*. *kivuensis*, *H*. *lateralis* and *H*. *viridiflavus*. Age class 0-LAGs individuals of these species had indeed SVL ranging from the size at metamorphosis to the size of 1 LAG old mature specimens (Fig 6). As the duration of growth period of the larger age class 0-LAGs individuals was an unknown fraction of a year, they were excluded from growth model estimation. Asymptotic maximum SVL_max_ as estimated by the von Bertalanffy model was significantly female-biased in *H*. *castaneus* and in *H*. *glandicolor* (assumed size at metamorphosis 14mm as in the closely related *H*. *viridiflavus*), whereas the growth coefficient k did not differ significantly (Table 2; comparison of CI, P<0.05). There were also significant differences among species with respect to SVL_max_ of males: *H*. *lateralis* < *H*. *castaneus* = *H*. *glandicolor* ≤ *H*. *kivuensis* = *H*. *viridiflavus* (Table 2; comparison of CI, P<0.05).

**Table 2:** Sex- and population-specific von Bertalanffy growth models for *Hyperolius* spp. n.s. means not significantly distinct from zero.

Species	sex	SVL_max_ (mm)	SVL-CI_95%_ (mm)	k	k-CI_95%_
*H*. *castaneus*	♂	24.7	23.4–26.0	2.398	1.166–3.619
*H*. *castaneus*	♀	29.6	27.6–31.6	2.831	0.516–5.147
*H*. *glandicolor*	♂	24.3	20.5–28.0	2.268	n.s.
*H*. *glandicolor*	♀	30.9	28.2–33.6	3.980	n.s.
*H*. *kivuensis*	♂	28.2	27.0–29.3	2.710	1.380–4.039
*H*. *lateralis*	♂	20.7	19.1–22.4	5.784	n.s.
*H*. *viridiflavus*	♂	28.2	26.1–30.3	2.409	0.833–3.986

### Demographic life-history traits in tropical anurans: A meta-analysis

Age at maturity of tropical anuran species varied between 0 (e.g. *H*. *lateralis*; this study, S1 Table) and 4 LAGs (*Boophis occidentalis*) [29] and did not differ significantly between males (n = 44 species) and females (n = 30 species, median: 1 LAG; Mann-Whitney W-test, W = 79.0, P = 0.1640). Minimum size at maturity ranged between 10mm SVL (*Nimbaphrynoides occidentalis*, *Mantella crocea*) and 128mm SVL (*Leptodactylus fallax*; S1 Table) [7, 30]. Medians of SVL at maturity did not differ significantly between males (25.2mm) and females (35.3mm; Mann-Whitney W-test, W = 712.5, P = 0.1371). Longevity varied between 1 (*H*. *lateralis*, this study) and 13 LAGs (*Euphlyctis hexadactylus*) [31] and did not differ significantly between males and females (median: 4 LAGs and 5 LAGs, respectively; Mann-Whitney W-test, W = 709.0, P = 0.3246). Maximum size observed ranged between 19.5mm SVL (*M*. *crocea*) [30] and 280mm SVL (*L*. *fallax*) [7]. Medians of maximum SVL did not differ significantly between males (33.9mm) and females (44.7mm; Mann-Whitney W-test, W = 776.0, P = 0.0809).

**Fig 7:**
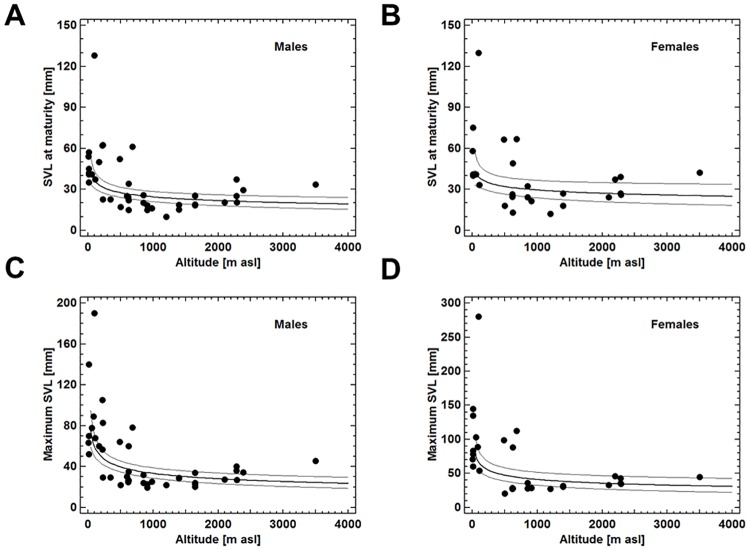
Multiplicative regression models describing the association between the size-related life-history traits and altitude, the only environmental variable explaining a significant amount of variance. Statistical details are given in the text. Each dot represents a distinct species of tropical anurans.

Factorial analysis of the data set including four demographic life-history traits of males pertaining to 41 species and latitude and altitude as proxies for local macroclimate yielded two principal components (eigenvalue > 1) which explained 72.6% of total variation (Table 3A). Subsequent VARIMAX-rotation showed that factor 1 was significantly loaded by the SVL traits and latitude and altitude, whereas factor 2 was loaded mainly by the two age traits. Multiple regression analysis demonstrated that only altitude explained a significant portion of variation the size variables. SVL at maturity and maximum SVL, respectively, correlated negatively with altitude (R^2^ = 0.331, F_1,41_ = 19.8, P<0.0001; R^2^ = 0.483, F_1,42_ = 38.3, P<0.0001). The variation explained was largest using multiplicative regression models (*SVL*_*maturity*_ = *e*^(4.394–0.173 * *in*(*altitude*))^; *SVL*_*max*_ = *e*^(5.244–0.251 * *in*(*altitude*))^; Fig 7).

**Table 3:** Factorial analyses of four demographic life-history traits of tropical anurans and corresponding latitude and altitude of collection sites. Matrix of factorial loads following VARIMAX-rotation of principal components in **(A)** Males (n = 41 species) and **(B)** Females (n = 28 species). Details on species involved are listed in S1 Table.

**(A) Males**				
	**Factor 1**	**Factor 2**	**Estimated communality**	**Specific variance**
**SVL at maturity**	0.667	0.573	0.774	0.226
**Maximum SVL**	0.786	0.482	0.850	0.150
**Age at maturity**	-0.161	0.907	0.848	0.152
**Longevity**	0.256	0.748	0.627	0.374
**Abs(Latitude)**	0.812	-0.275	0.735	0.265
**Altitude**	-0.718	-0.108	0.527	0.473
**(B) Females**				
	**Factor 1**	**Factor 2**	**Estimated communality**	**Specific variance**
**SVL at maturity**	0.883	0.303	0.873	0.127
**Maximum SVL**	0.787	0.501	0.870	0.129
**Age at maturity**	0.883	-0.385	0.928	0.072
**Longevity**	0.707	0.223	0.550	0.450
**Abs(Latitude)**	0.240	0.721	0.577	0.422
**Altitude**	-0.024	-0.884	0.783	0.217

The analogous analysis of the data on females pertaining to 24 species yielded similar results as in the males. Two principal components (eigenvalue > 1) explained 76.3% of total variation (Table 3B). Factor loading by the original variables of the data set was as described for males. Multiple regression analysis demonstrated that only altitude explained a significant portion of variation the size variables. Again, there were significant correlations between SVL at maturity and maximum SVL, respectively, and altitude (R^2^ = 0.255, F_1,24_ = 9.23, P = 0.0059; R^2^ = 0.284, F_1,24_ = 10.5, P = 0.0036), described by multiplicative regression models (*SVL*_*maturity*_ = *e*^(4.550–0.156 * *in*(*altitude*))^; *SVL*_*max*_ = *e*^(5.231–0.213 * *in*(*altitude*))^; Fig 7).

## Discussion

All *Hyperolius* spp. surveyed in this study included individuals with one to four LAGs demonstrating that there are periods of arrested bone growth in frogs of the tropical mountains of East Africa. We critically evaluate evidence for the assumed annual periodicity of LAG formation and continue with a review on the variation of age- and SVL-related life-history traits observed in tropical and temperate-zone anuran species.

### Is skeletochronology reliable for aging tropical anurans?

Afromontane anurans showed regular alternations between periods of periosteal growth and arrestment of growth analogous to those observed in temperate-zone amphibians. Bone growth was observed in all individuals collected during the rainy season (e.g. Fig 2). Our data suggest that reinforcement of the genetically-based circannual growth rhythm is mediated by the seasonal variation of precipitation rather than that of temperature. Seven out of nine *H*. *castaneus* specimens held in captivity (equivalent to a prolonged dry period) had formed an additional LAG as predicted (77.8%), whereas the bones of the deviant two individuals did not grow during the whole period of captivity (presumably quality and quantity of food were suboptimal). Similarly, 11 out 21 captive-held *L*. *fallax* showed the predicted number of additional LAGs (52%), the other one supernumerary LAG or one or two less than predicted [7] supporting the circannual periodicity of LAG formation even in the zoo environment [32]. In the natural habitat LAG formation during the dry period was also observed in the Asian frog *Sylvirana nigrovittata* [12] emphasising that low water availability combined with optimal temperatures may act as external zeitgeber for the circannual clock in the same way as hot temperatures and dryness in arid regions and cold temperatures in temperate climate zones (4). There is no indication that skeletochronological age estimation may be generally unreliable in tropical amphibians because LAG formation is less pronounced as in the temperate zone ([7]; but see [2] for a failure of LAG detection in perennial *Litoria lesueuri*). The rate of correct skeleotochronological age estimation is about 86% in temperate-zone amphibians younger than eight years [4] and available evidence for tropical anurans suggests that the rate is similar. We conclude that aging tropical frogs by counting LAGs yields a conservative estimate of longevity because in case of proven deviation from the actual lifespan longevity tends to be underestimated ([2, 7] this study). Unlike adults of temperate-zone anurans, many reproductive adults of tropical species do not show visible LAGs because sexual maturity is often reached before finishing the first year of life ([13, 14, 33] this study).

### Does variation of demographic life-history traits differ among tropical and temperate anurans?

#### Age at maturity

Tropical anurans mature on average one year earlier than temperate-zone species (estimate based on compiled published data of 124 species). Temperature- and precipitation regime in the tropics usually allow for activity during most of the year so that the majority of species mature within their first or second year of life (e.g. Table 1), while the delayed maturity of temperate-zone anurans can be attributed to the shorter annual growth period. Morrison and Hero [1] predict that age at maturity generally increases along latitudinal and altitudinal clines. Case studies on species inhabiting wide geographical ranges provide support for this prediction in temperate-zone anurans [5, 6]. In contrast, we did not find any evidence that age at maturity is affected by latitudinal or altitudinal variation in tropical species. Yet, the age variation detectable by skeletochronology has a resolution of one year, whereas a resolution of months would have been required possibly to identify potential latitudinal and altitudinal effects. We cannot completely rule out clinal geographical influence on the age at maturity in tropical anurans, but we expect adaptive delay of maturation to be rare because of the generally favourable environmental conditions.

#### Minimum size at maturity

The tendency of females being larger than males at attaining maturity was not statistically significant in the complete data set on tropical species, but well established in two *Hyperolius* species (see Table 2). Comparing median SVL at maturity of tropical anuran species (26.9mm) with that of temperate ones (41.8mm; estimate based on compiled published data of 18 species) the size threshold of maturity seems to be considerably lower in the tropics. Since female size is positively related to clutch size in most anuran species [34, 35] and longevity of tropical species is low ([17, 30], this study), lifetime fecundity appears to be much smaller than in temperate-zone species, paralleling trait evolution in birds [36]. At the same time, the diversity of reproductive modes including parental care is far greater in Amazonian amphibians [37] than in those of temperate regions [38] suggesting a potential evolutionary advantage of k-strategists with small clutches in the Neotropics. Yet, the eight *Hyperolius* spp. analysed in this study and all other Rwandan anuran species are short-lived and unspecialized pond or stream breeders [20, 23] demonstrating that the coupling between low lifetime fecundity and parental care in the Neotropics does not prevail in the Afrotropics. It is intriguing that size of recently matured tropical anurans decreases with increasing altitude, a factor explaining about a third of observed variance. Since the surface/volume-ratio is unfavourable for small individuals with respect to evaporative water loss and thermal relations [39], this tendency may indicate a trade-off between early maturation and size.

#### Longevity

The maximum lifespan of tropical anurans is about 2–3 years lower than that of temperate anurans (estimate based on compiled published data of 140 species). In long-lived species the discrepancy is even greater, 13 LAGs in the tropical *E*. *hexadactylus* [31] compared with 17 LAGs in the temperate *E*. *calamita* [40] and 18 LAGs in *R*. *temporaria* [41]. This pattern seems to indicate that the risk of dying during the inactivity period of winter is probably lower than that of being predated during the season of activity. Again, longevity is predicted to increase along latitudinal and altitudinal clines [1]. There is ample support for this prediction in temperate-zone amphibians [3, 5, 6, 42, 43]. In contrast, longevity of tropical species did not co-vary significantly with latitude or altitude suggesting local constraints such as predator impact and parasite load being more important than macroclimate gradients.

#### Maximum size

Short lifespan and small maximum size are associated in most tropical species (e.g. Fig 5 for *Hyperolius* spp.). Maximum SVL (median: 33.2mm) is only about half of that of temperate-zone species (median: 65.0mm; estimate based on compiled published data of 80 species), but there are remarkable exceptions especially in aquatic tropical species (e.g. SVL up to 320mm in *Conraua goliath* [44] and 170.3mm in *Telmatobius macrostomus* [45]). Maximum SVL of European and North American anuran species increases with latitude [46], whereas the intraspecific pattern is more complex and SVL decreases with altitude [5, 47]. The later tendency is present in tropical frogs as well, whereas latitudinal effects seem to be absent. Female size variation in temperate-zone species has been suggested to be the evolutionary by-product of the optimization of lifetime fecundity [5, 47], while that in tropical species remains enigmatic requiring further investigations.

## Conclusions

Variations of demographic life-history traits in afromontane *Hyperolius* spp. are in line with those in anuran species inhabiting the tropical regions in South America, Madagascar and Asia. Common features are early sexual maturation at small size and low longevity resulting in low lifetime fecundity. This pattern contrasts with that found in temperate-zone anurans which mature later at larger size and grow considerably older, experiencing greater lifetime fecundity. Macroclimatic constraints mediated by latitude and altitude account for a large portion of age- and size-related life-history traits in temperate-zone species, whereas only size-related traits co-vary slightly with altitude and not at all with latitude in tropical species. We conclude that the contraction of activity period at increasing latitudes and altitudes shapes demographic life-history traits of anurans in the temperate region. In the tropical belt, however, climate does not constrain activity in the lowland at any latitude and only to a minor extent in the highlands indicating that timing of sexual maturation, short lifespan and size limitation respond to different evolutionary forces than those in the temperate zone.

## Supporting information

S1 Table List of 44 tropical species and corresponding references used for the meta-analysis of demographic life-history traits. (DOCX)

S2 Table Original data set on age, size, gender and geographical origin of specimens pertaining to eight *Hyperolius* species. (DOCX)

S3 Table Data extracted from references listed in S1 Table on age, size, gender and geographical origin of tropical anuran specimens. This data set was used for the metaanalysis.
(DOCX)

S4 Table References used for the meta-analysis of demographic life-history traits, but not listed in the main text. (DOCX)
