# Supplementary material for: Tropical anurans mature early and die young: Evidence from eight Afromontane Hyperolius species and a meta-analysis
Source: PLoS One. 2017 Feb 9;12(2):e0171666. doi: 10.1371/journal.pone.0171666 (PMC5300166; doi:10.1371/journal.pone.0171666)
Supplement: S1 Table — (DOCX) [file pone.0171666.s001.docx]

**S1 Table.** List of 44 tropical species and corresponding references used for the meta-analysis of demographic life-history traits

| **Species** | **Locality**  **(latitude/altitude)** | **Data on males** | **Data on females** | **References** |
| --- | --- | --- | --- | --- |
| *Atelopus chiriquiensis* | 9.18°N / 2,200m |  | x | 16 |
| *Atelopus peruensis* | 5.5°N / 3,500m | x | x | 16 |
| *Atelopus* spec. | 9.3°N / 600m | x |  | 16 |
| *Boophis occidentalis* | 14.3°S / 170m | x |  | 29 |
| *Boophis williamsi* | 19.35°S / 2,285m | x | x | 48 |
| *Bufo bankorensis* | 22.05°N / 230m | x |  | 13 |
| *Cophixalus ornatus* | 19.00°S / 980m | x |  | 59 |
| *Duttaphrynus melanostictus* | 20.3°N / 56m | x | x | 49, 63 |
| *Dyscophus antongilii* | 16.85°S / 486m | X | x | 33 |
| *Dyscophus guineti* | 18.7°S / 680m | x | X | 50 |
| *Euphlyctis cyanophlyctis* | 15.3°N / 626m | x | x | 13, 51, 52, 53 |
| *Euphlyctis cyanophlyctis* | 21.68°N / 14m | x | x | 63 |
| *Euphlyctis hexadactylus* | 21.68°N / 14m | x | x | 31, 63 |
| *Fejervarya cancrivora* | 6.7°S / 200m | x |  | 14 |
| *Fejervarya limnocharis* | 15.3°N / 626m | x |  | 54 |
| *Hoplobatrachus tigerinus* | 14.8°N / 11m | x | x | 64, 65, 66 |
| *Hyperolius castaneus* | 2.4°S / 2,100 | x | x | This study |
| *Hyperolius* cf. *cinnamomeoventris* | 2.6°S / 1,643m | x |  | This study |
| *Hyperolius discodactylus* | 2.5°S / 2,389m | x |  | This study |
| *Hyperolius glandicolor* | 1.6°S / 2,287m | x | x | This study |
| *Hyperolius kivuensis* | 2.6°S / 1,643m | x |  | This study |
| *Hyperolius lateralis* | 2.6°S / 1,643m | x |  | This study |
| *Hyperolius rwandae* | 2.6°S / 1,643m | X |  | This study |
| *Hyperolius viridiflavus* | 2.6°S / 1,643m | x |  | This study |
| *Hypsiboas rosenbergi* | 9.09°N / 87m | x | x | 60 |
| *Leptodactylus fallax* | 16.75°N / 100m | x | x | 7 |
| *Limnonectes macrodon* | 6.7°S / 220m | x |  | 14 |
| *Mantella baroni* | 20.8°S / 1,400m | x | x | 17, 18, 30 |
| *Mantella bernhardi* | 21.4°S / 500m | x | x | 18, 30 |
| *Mantella cowani* | 20.8°S / 1,400m | x | x | 17, 18 |
| *Mantella crocea* | 18.35°S / 910m | x |  | 18 |
| *Mantella expectata* | 22.16°S / 850m | x | x | 55 |
| *Mantella laevigata* | 15.3°S / 615m | x | x | 18 |
| *Mantella nigricans* | 15.3°S / 615m | x | x | 18 |
| *Mantella pulchra* | 18.35°S / 910m | x | x | 18 |
| *Mantidactylus pauliani* | 19.35°S / 2,285 | x | x | 48 |
| *Microhyla ornata* | 15.3°N / 626m | x | x | 56, 63 |
| *Micryletta steinegeri* | 22.1°N / 230m | x |  | 13 |
| *Nimbaphrynoides occidentalis* | 7.6°N / 1200 m | x | x | 61 |
| *Phrynobatrachus guineensis* | 5.8°N / 200m | x | x | 62 |
| *Polypedates maculatus* | 21.68°N / 14m | x | x | 57, 58, 63 |
| *Polypedates teraiensis* | 21.68°N / 14m | x | x | 63 |
| *Scaphiophryne gottlebei* | 22.16°S / 850m | x | x | 55 |
| *Sclerophrys pentoni* | 18.45°N / 8m | X | X | 19 |
| *Sylvirana nigrovittata* | 13.4°N / 110m | x | x | 12 |
