# Supplementary material for: Tropical anurans mature early and die young: Evidence from eight Afromontane Hyperolius species and a meta-analysis
Source: PLoS One. 2017 Feb 9;12(2):e0171666. doi: 10.1371/journal.pone.0171666 (PMC5300166; doi:10.1371/journal.pone.0171666)
Supplement: S2 Table — (DOCX) [file pone.0171666.s002.docx]

**S2 Table.** Original data set on age, size, gender and geographical origin of specimens pertaining to eight *Hyperolius* species.

| Id_Nr | Genus | Species | Latitude | Longitude | Elevation | SVL | sex | ML | LAGs |
| --- | --- | --- | --- | --- | --- | --- | --- | --- | --- |
|  |  |  | [°] | [°] | [m asl] | [mm] | 1=male, 2=female | 0=absent, 1=present |  |
| 1I | *Hyperolius* | *glandicolor* | -1,64378 | 29,39784 | 2287 | 27 | 1 |  | 2 |
| 2I | *Hyperolius* | *glandicolor* | -1,64378 | 29,39784 | 2287 | 31 | 2 | 1 | 2 |
| 3I | *Hyperolius* | *glandicolor* | -1,64378 | 29,39784 | 2287 | 33 | 2 | 1 | 2 |
| 4I | *Hyperolius* | *glandicolor* | -1,64378 | 29,39784 | 2287 | 23 | 1 | 1 | 1 |
| 5I | *Hyperolius* | *glandicolor* | -1,64378 | 29,39784 | 2287 | 23,5 | 1 | 1 | 1 |
| 6I | *Hyperolius* | *glandicolor* | -1,64378 | 29,39784 | 2287 | 24 | 1 | 1 | 1 |
| 7I | *Hyperolius* | *glandicolor* | -1,64378 | 29,39784 | 2287 | 31 | 2 | 0 | 2 |
| 8I | *Hyperolius* | *glandicolor* | -1,64378 | 29,39784 | 2287 | 33 | 2 | 1 | 1 |
| 9I | *Hyperolius* | *glandicolor* | -1,64378 | 29,39784 | 2287 | 31 | 2 |  |  |
| 10I | *Hyperolius* | *glandicolor* | -1,64378 | 29,39784 | 2287 | 23,5 | 1 | 1 | 2 |
| 11I | *Hyperolius* | *glandicolor* | -1,64378 | 29,39784 | 2287 | 22 | 1 | 1 | 2 |
| 12I | *Hyperolius* | *glandicolor* | -1,64378 | 29,39784 | 2287 | 32 | 2 | 1 | 1 |
| 13I | *Hyperolius* | *glandicolor* | -1,64378 | 29,39784 | 2287 | 30 | 2 | 0 | 2 |
| 14I | *Hyperolius* | *glandicolor* | -1,64378 | 29,39784 | 2287 | 28 | 2 | 1 | 2 |
| 16I | *Hyperolius* | *glandicolor* | -1,64378 | 29,39784 | 2287 | 28 | 2 | 1 | 1 |
| 17I | *Hyperolius* | *glandicolor* | -1,64378 | 29,39784 | 2287 | 26 | 2 | 1 | 1 |
| 18I | *Hyperolius* | *glandicolor* | -1,64378 | 29,39784 | 2287 | 32 | 2 | 1 | 2 |
| 19I | *Hyperolius* | *glandicolor* | -1,64378 | 29,39784 | 2287 | 27 | 1 | 1 | 1 |
| 638 | *Hyperolius* | *glandicolor* | -1,441667 | 29,494444 | 2879 | 34,49 | 2 | 1 | 1 |
| 642 | *Hyperolius* | *glandicolor* | -1,64378 | 29,39784 | 2287 | 21,97 | 1 | 1 | 1 |
| 643 | *Hyperolius* | *glandicolor* | -1,64378 | 29,39784 | 2287 | 20,48 | 1 | 1 | 1 |
| 644 | *Hyperolius* | *glandicolor* | -1,64378 | 29,39784 | 2287 | 34,85 | 2 | 1 | 1 |
| 645 | *Hyperolius* | *glandicolor* | -1,64378 | 29,39784 | 2287 | 32,01 | 2 | 1 | 1 |
| 647 | *Hyperolius* | *glandicolor* | -1,64378 | 29,39784 | 2287 | 17,98 | 1 | 1 | 0 |
| 648 | *Hyperolius* | *glandicolor* | -1,64378 | 29,39784 | 2287 | 22,54 | 1 | 1 | 1 |
| 691 | *Hyperolius* | *glandicolor* | -1,48557 | 29,88928 | 2055 | 32,04 | 2 | 1 | 1 |
|  |  |  |  |  |  |  |  |  |  |
| 530 | *Hyperolius* | *viridiflavus* | -2,607 | 29,757 | 1643 | 29,65 | 1 | 1 | 1 |
| 532 | *Hyperolius* | *viridiflavus* | -2,607 | 29,757 | 1643 | 30,95 | 1 | 1 | 1 |
| 533 | *Hyperolius* | *viridiflavus* | -2,607 | 29,757 | 1643 | 28,38 | 1 | 1 | 1 |
| 564 | *Hyperolius* | *viridiflavus* | -1,826 | 30,266 | 1437 | 25,84 | 1 | 1 | 1 |
| 565 | *Hyperolius* | *viridiflavus* | -1,826 | 30,266 | 1437 | 25,22 | 1 | 1 | 0 |
| 579 | *Hyperolius* | *viridiflavus* | -1,50729 | 29,65321 | 1807 | 29,7 | 1 | 1 | 0 |
| 613 | *Hyperolius* | *viridiflavus* | -2,09843 | 29,77989 | 1785 | 31,43 | 1 | 1 | 1 |
| 615 | *Hyperolius* | *viridiflavus* |  |  |  | 33,72 | 1 | 1 | 1 |
| 617 | *Hyperolius* | *viridiflavus* |  |  |  | 29,85 | 1 | 1 | 1 |
| 623 | *Hyperolius* | *viridiflavus* | -1,72216 | 29,63664 | 1457 | 31,3 | 1 | 1 | 2 |
| 629 | *Hyperolius* | *viridiflavus* | -2,45208 | 29,45824 | 2006 | 27,97 | 1 | 0 | 1 |
| 630 | *Hyperolius* | *viridiflavus* | -2,45208 | 29,45824 | 2006 | 28,9 | 1 | 1 | 1 |
| 636 | *Hyperolius* | *viridiflavus* | -2,5766 | 29,59607 | 1518 | 32,76 | 1 | 1 | 2 |
| 713 | *Hyperolius* | *viridiflavus* | -2,20525 | 30,2717 | 1324 | 26,59 | 1 | 1 | 1 |
| 721 | *Hyperolius* | *viridiflavus* | -2,46883 | 29,68242 | 1580 | 25,59 | 1 | 0 |  |
| 1 | *Hyperolius* | *viridiflavus* | -2,607 | 29,757 | 1643 | 29,5 | 1 | 1 |  |
| 2 | *Hyperolius* | *viridiflavus* | -2,607 | 29,757 | 1643 | 31,5 | 1 | 1 | 2 |
| 3 | *Hyperolius* | *viridiflavus* | -2,607 | 29,757 | 1643 | 28,5 | 1 | 1 | 1 |
| 4 | *Hyperolius* | *viridiflavus* | -2,607 | 29,757 | 1643 | 30 | 1 | 1 | 1 |
| 5 | *Hyperolius* | *viridiflavus* | -2,607 | 29,757 | 1643 | 30 | 1 | 1 | 1 |
| 6 | *Hyperolius* | *viridiflavus* | -2,607 | 29,757 | 1643 | 32,5 | 1 | 1 | 1 |
| 7 | *Hyperolius* | *viridiflavus* | -2,607 | 29,757 | 1643 | 28,5 | 1 | 1 | 1 |
| V1 | *Hyperolius* | *viridiflavus* | -2,607 | 29,757 | 1643 | 22,7 | 1 | 0 | 1 |
| V2 | *Hyperolius* | *viridiflavus* | -2,607 | 29,757 | 1643 | 25 | 1 | 0 | 1 |
| V3 | *Hyperolius* | *viridiflavus* | -2,607 | 29,757 | 1643 | 23 | 1 | 1 | 2 |
| V4 | *Hyperolius* | *viridiflavus* | -2,607 | 29,757 | 1643 | 26,3 | 1 | 1 | 1 |
| V5 | *Hyperolius* | *viridiflavus* | -2,607 | 29,757 | 1643 | 23,9 | 1 | 1 | 2 |
| V6 | *Hyperolius* | *viridiflavus* | -2,607 | 29,757 | 1643 | 22,1 | 1 | 1 | 1 |
| V7 | *Hyperolius* | *viridiflavus* | -2,607 | 29,757 | 1643 | 29 | 1 | 0 | 1 |
| V8 | *Hyperolius* | *viridiflavus* | -2,607 | 29,757 | 1643 | 28,4 | 1 | 0 | 1 |
| V9 | *Hyperolius* | *viridiflavus* | -2,607 | 29,757 | 1643 | 23,7 | 1 | 0 | 2 |
| V10 | *Hyperolius* | *viridiflavus* | -2,607 | 29,757 | 1643 | 26,55 | 1 | 1 | 1 |
| V11 | *Hyperolius* | *viridiflavus* | -2,607 | 29,757 | 1643 | 24,8 | 1 | 1 | 1 |
| V12 | *Hyperolius* | *viridiflavus* | -2,607 | 29,757 | 1643 | 26,7 | 1 | 1 | 1 |
| V13 | *Hyperolius* | *viridiflavus* | -2,607 | 29,757 | 1643 | 25,8 | 1 | 0 | 1 |
| V14 | *Hyperolius* | *viridiflavus* | -2,607 | 29,757 | 1643 | 25,35 | 1 | 0 | 1 |
| V15 | *Hyperolius* | *viridiflavus* | -2,607 | 29,757 | 1643 | 23,9 | 1 | 1 | 1 |
| V16 | *Hyperolius* | *viridiflavus* | -2,607 | 29,757 | 1643 | 23,5 | 1 | 0 | 1 |
| V17 | *Hyperolius* | *viridiflavus* | -2,607 | 29,757 | 1643 | 28,4 | 2 | 1 | 1 |
| V18 | *Hyperolius* | *viridiflavus* | -2,607 | 29,757 | 1643 | 22,2 | 1 | 0 | 2 |
| V19 | *Hyperolius* | *viridiflavus* | -2,607 | 29,757 | 1643 | 22,8 | 1 | 0 | 1 |
| V20 | *Hyperolius* | *viridiflavus* | -2,607 | 29,757 | 1643 | 26 | 1 | 0 | 1 |
| V21 | *Hyperolius* | *viridiflavus* | -2,607 | 29,757 | 1643 | 27,1 | 1 | 1 | 1 |
| V22 | *Hyperolius* | *viridiflavus* | -2,607 | 29,757 | 1643 | 23,2 | 1 | 1 | 2 |
| V24 | *Hyperolius* | *viridiflavus* | -2,607 | 29,757 | 1643 | 25,2 | 1 | 0 | 1 |
| V25 | *Hyperolius* | *viridiflavus* | -2,607 | 29,757 | 1643 | 27,9 | 1 | 1 | 1 |
| V_1 | *Hyperolius* | *viridiflavus* | -2,607 | 29,757 | 1643 | 23,9 | 1 | 1 | 1 |
| V_2 | *Hyperolius* | *viridiflavus* | -2,607 | 29,757 | 1643 | 23,8 | 1 | 1 | 1 |
|  |  |  |  |  |  |  |  |  |  |
| 1S | *Hyperolius* | *spec (cinnamomeoventris-group)* | -2,607 | 29,757 | 1643 | 21,35 | 1 | 0 | 3 |
| 2S | *Hyperolius* | *spec (cinnamomeoventris-group)* | -2,607 | 29,757 | 1643 | 22,46 | 1 | 0 | 1 |
| 3S | *Hyperolius* | *spec (cinnamomeoventris-group)* | -2,607 | 29,757 | 1643 | 22,54 | 1 | 0 | 1 |
| 4S | *Hyperolius* | *spec (cinnamomeoventris-group)* | -2,607 | 29,757 | 1643 | 21,57 | 1 | 0 | 2 |
| 5S | *Hyperolius* | *spec (cinnamomeoventris-group)* | -2,607 | 29,757 | 1643 | 20,9 | 1 | 0 | 2 |
| 7S | *Hyperolius* | *spec (cinnamomeoventris-group)* | -2,607 | 29,757 | 1643 | 21,28 | 1 | 0 | 2 |
| 8S | *Hyperolius* | *spec (cinnamomeoventris-group)* | -2,607 | 29,757 | 1643 | 19,92 | 1 | 1 | 2 |
| 635 | *Hyperolius* | *spec (cinnamomeoventris-group)* | -2,5766 | 29,59607 | 1518 | 18,62 | 1 | 0 | 1 |
| 651 | *Hyperolius* | *spec (cinnamomeoventris-group)* | -2,607 | 29,757 | 1643 | 22,11 | 1 | 1 | 1 |
| 1 | *Hyperolius* | *spec (cinnamomeoventris-group)* | -2,607 | 29,757 | 1643 | 19,2 | 1 | 0 | 1 |
| 2 | *Hyperolius* | *spec (cinnamomeoventris-group)* | -2,607 | 29,757 | 1643 | 21,2 | 1 | 0 | 1 |
| 3 | *Hyperolius* | *spec (cinnamomeoventris-group)* | -2,607 | 29,757 | 1643 | 22,09 | 1 | 0 | 1 |
|  |  |  |  |  |  |  |  |  |  |
| 11S | *Hyperolius* | *rwandae* | -2,607 | 29,757 | 1643 | 19,29 | 1 | 0 | 2 |
| 12S | *Hyperolius* | *rwandae* | -2,607 | 29,757 | 1643 | 20,23 | 1 | 0 | 1 |
| 13S | *Hyperolius* | *rwandae* | -2,607 | 29,757 | 1643 | 18,16 | 1 | 0 | 1 |
| 14S | *Hyperolius* | *rwandae* | -2,607 | 29,757 | 1643 | 19,43 | 1 | 0 | 4 |
| 15S | *Hyperolius* | *rwandae* | -2,607 | 29,757 | 1643 | 19,9 | 1 | 0 | 2 |
| 16S | *Hyperolius* | *rwandae* | -2,607 | 29,757 | 1643 | 19,07 | 1 | 0 | 2 |
| 17S | *Hyperolius* | *rwandae* | -2,607 | 29,757 | 1643 | 18,55 | 1 | 0 | 2 |
| 9S | *Hyperolius* | *rwandae* | -2,607 | 29,757 | 1643 | 23,54 | 2 | 1 | 1 |
| 10S | *Hyperolius* | *rwandae* | -2,607 | 29,757 | 1643 | 23,46 | 2 | 1 | 2 |
|  |  |  |  |  |  |  |  |  |  |
| 567 | *Hyperolius* | *discodactylus* | -1,82325 | 29,36045 | 2086 | 40 | 2 | 1 | 1 |
| 568 | *Hyperolius* | *discodactylus* | -1,82325 | 29,36045 | 2086 | 33,18 | 1 | 1 | 1 |
| 606 | *Hyperolius* | *discodactylus* | -2,48052 | 29,22847 | 2283 | 34,44 | 1 | 1 | 1 |
| 632 | *Hyperolius* | *discodactylus* | -2,528683 | 29,354033 | 2379 | 31,05 | 1 | 1 | 2 |
| 633 | *Hyperolius* | *discodactylus* | -2,528683 | 29,354033 | 2379 | 32,14 | 1 | 1 | 1 |
| 724 | *Hyperolius* | *discodactylus* | -2,4478 | 29,10724 | 1813 | 29,44 | 1 | 1 | 1 |
|  |  |  |  |  |  |  |  |  |  |
| 1lat | *Hyperolius* | *lateralis* | -2,45208 | 29,45824 | 2006 | 20 | 1 | 1 | 1 |
| 2lat | *Hyperolius* | *lateralis* | -2,45208 | 29,45824 | 2006 | 20 | 1 | 1 | 1 |
| 3lat | *Hyperolius* | *lateralis* | -2,45208 | 29,45824 | 2006 | 20 | 1 | 1 | 1 |
| 6S | *Hyperolius* | *lateralis* | -2,607 | 29,757 | 1643 | 22,34 | 1 | 1 | 1 |
| 646 | *Hyperolius* | *lateralis* | -2,607 | 29,757 | 1643 | 21,66 | 1 | 1 | 1 |
| 650 | *Hyperolius* | *lateralis* | -2,607 | 29,757 | 1643 | 21,24 | 1 | 1 | 0 |
| 652 | *Hyperolius* | *lateralis* | -2,607 | 29,757 | 1643 | 22 | 1 | 1 | 0 |
| 653 | *Hyperolius* | *lateralis* | -2,607 | 29,757 | 1643 | 22,14 | 1 | 1 | 0 |
| 654 | *Hyperolius* | *lateralis* | -2,607 | 29,757 | 1643 | 23,14 | 1 | 1 | 0 |
| 655 | *Hyperolius* | *lateralis* | -2,607 | 29,757 | 1643 | 19,91 | 1 | 1 | 0 |
| 660 | *Hyperolius* | *lateralis* | -2,607 | 29,757 | 1643 | 20,91 | 1 | 1 | 0 |
| 661 | *Hyperolius* | *lateralis* | -2,607 | 29,757 | 1643 | 21,07 | 1 | 1 | 0 |
| 662 | *Hyperolius* | *lateralis* | -2,607 | 29,757 | 1643 | 20,09 | 1 | 1 | 0 |
| 663 | *Hyperolius* | *lateralis* | -2,607 | 29,757 | 1643 | 23,17 | 1 | 1 | 0 |
| 664 | *Hyperolius* | *lateralis* | -2,607 | 29,757 | 1643 | 19,03 | 1 | 1 | 1 |
| 665 | *Hyperolius* | *lateralis* | -2,45208 | 29,45824 | 2006 | 20,03 | 1 | 1 | 1 |
| 666 | *Hyperolius* | *lateralis* | -2,45208 | 29,45824 | 2006 | 21,28 | 1 | 1 | 1 |
| 667 | *Hyperolius* | *lateralis* | -2,45208 | 29,45824 | 2006 | 22,14 | 1 | 1 | 0 |
| 669 | *Hyperolius* | *lateralis* | -2,45208 | 29,45824 | 2006 | 19,18 | 1 | 1 | 1 |
| 710 | *Hyperolius* | *lateralis* | -2,20525 | 30,2717 | 1324 | 23,93 | 1 | 1 | 0 |
| L1 | *Hyperolius* | *lateralis* | -2,607 | 29,757 | 1643 | 19,7 | 1 | 1 | 0 |
| L2 | *Hyperolius* | *lateralis* | -2,607 | 29,757 | 1643 | 20,7 | 1 | 1 | 1 |
| L3 | *Hyperolius* | *lateralis* | -2,607 | 29,757 | 1643 | 20,1 | 1 | 1 | 1 |
| L4 | *Hyperolius* | *lateralis* | -2,607 | 29,757 | 1643 | 19,7 | 1 | 1 | 0 |
| L5 | *Hyperolius* | *lateralis* | -2,607 | 29,757 | 1643 | 20,4 | 1 | 1 | 0 |
| L6 | *Hyperolius* | *lateralis* | -2,607 | 29,757 | 1643 | 20 | 1 | 1 | 1 |
| L7 | *Hyperolius* | *lateralis* | -2,607 | 29,757 | 1643 | 21,1 | 1 | 1 | 0 |
| L8 | *Hyperolius* | *lateralis* | -2,607 | 29,757 | 1643 | 20,6 | 1 | 1 | 0 |
| L9 | *Hyperolius* | *lateralis* | -2,607 | 29,757 | 1643 | 19,3 | 1 | 1 | 0 |
| L10 | *Hyperolius* | *lateralis* | -2,607 | 29,757 | 1643 | 20,4 | 1 | 1 | 0 |
| L11 | *Hyperolius* | *lateralis* | -2,607 | 29,757 | 1643 | 20 | 1 | 1 | 0 |
| L12 | *Hyperolius* | *lateralis* | -2,607 | 29,757 | 1643 | 19,8 | 1 | 1 | 0 |
| L13 | *Hyperolius* | *lateralis* | -2,607 | 29,757 | 1643 | 21,2 | 1 | 1 | 0 |
| L14 | *Hyperolius* | *lateralis* | -2,607 | 29,757 | 1643 | 20,1 | 1 | 1 | 1 |
| L15 | *Hyperolius* | *lateralis* | -2,607 | 29,757 | 1643 | 20,1 | 1 | 1 | 1 |
| L16 | *Hyperolius* | *lateralis* | -2,607 | 29,757 | 1643 | 20,5 | 1 | 1 | 1 |
| L17 | *Hyperolius* | *lateralis* | -2,607 | 29,757 | 1643 | 20,2 | 1 | 1 | 0 |
| L18 | *Hyperolius* | *lateralis* | -2,607 | 29,757 | 1643 | 20,1 | 1 | 1 | 0 |
| L19 | *Hyperolius* | *lateralis* | -2,607 | 29,757 | 1643 | 20,9 | 1 | 1 | 0 |
| L20 | *Hyperolius* | *lateralis* | -2,607 | 29,757 | 1643 | 19,9 | 1 | 1 | 0 |
| L21 | *Hyperolius* | *lateralis* | -2,607 | 29,757 | 1643 | 21,9 | 1 | 1 | 0 |
| L22 | *Hyperolius* | *lateralis* | -2,607 | 29,757 | 1643 | 20,6 | 1 | 1 | 0 |
| L23 | *Hyperolius* | *lateralis* | -2,607 | 29,757 | 1643 | 20,1 | 1 | 1 | 0 |
| L24 | *Hyperolius* | *lateralis* | -2,607 | 29,757 | 1643 | 21,1 | 1 | 1 | 0 |
|  |  |  |  |  |  |  |  |  |  |
| 529 | *Hyperolius* | *kivuensis* | -2,607 | 29,757 | 1643 | 26,1 | 1 | 1 | 1 |
| 531 | *Hyperolius* | *kivuensis* | -2,607 | 29,757 | 1643 | 33,93 | 1 | 1 | 2 |
| 560 | *Hyperolius* | *kivuensis* | -2,607 | 29,757 | 1643 | 28,71 | 1 | 1 | 1 |
| 561 | *Hyperolius* | *kivuensis* | -2,607 | 29,757 | 1643 | 36,05 | 2 | 0 | 1 |
| 580 | *Hyperolius* | *kivuensis* | -1,50729 | 29,65321 | 1807 | 30,73 | 1 | 1 | 1 |
| 612 | *Hyperolius* | *kivuensis* | -2,09843 | 29,77989 | 1785 | 29,39 | 1 | 1 | 1 |
| 616 | *Hyperolius* | *kivuensis* |  |  |  | 31,49 | 1 | 0 | 1 |
| 628 | *Hyperolius* | *kivuensis* | -2,45208 | 29,45824 | 2006 | 31,05 | 1 | 1 | 2 |
| 637 | *Hyperolius* | *kivuensis* | -2,5766 | 29,59607 | 1518 | 33,39 | 1 | 0 | 3 |
| 709 | *Hyperolius* | *kivuensis* | -2,46883 | 29,68242 | 1580 | 25,42 | 1 | 1 | 0 |
| 714 | *Hyperolius* | *kivuensis* | -2,20525 | 30,2717 | 1324 | 26,65 | 1 | 0 | 0 |
| 715 | *Hyperolius* | *kivuensis* | -2,20525 | 30,2717 | 1324 | 28,5 | 1 | 1 | 2 |
| 720 | *Hyperolius* | *kivuensis* | -2,22441 | 30,82755 | 1292 | 25,75 | 1 | 1 | 0 |
| KK1 | *Hyperolius* | *kivuensis* | -2,607 | 29,757 | 1643 | 23,5 | 1 | 1 | 1 |
| KK2 | *Hyperolius* | *kivuensis* | -2,607 | 29,757 | 1643 | 29,2 | 1 | 0 | 1 |
| KK3 | *Hyperolius* | *kivuensis* | -2,607 | 29,757 | 1643 | 28,15 | 1 | 0 | 1 |
| KK4 | *Hyperolius* | *kivuensis* | -2,607 | 29,757 | 1643 | 28,7 | 1 | 0 | 1 |
| KK5 | *Hyperolius* | *kivuensis* | -2,607 | 29,757 | 1643 | 27,2 | 1 | 0 | 1 |
| KK6 | *Hyperolius* | *kivuensis* | -2,607 | 29,757 | 1643 | 31,2 | 1 | 0 | 2 |
| KK7 | *Hyperolius* | *kivuensis* | -2,607 | 29,757 | 1643 | 30,8 | 1 | 0 | 1 |
| KK8 | *Hyperolius* | *kivuensis* | -2,607 | 29,757 | 1643 | 26,3 | 1 | 0 | 2 |
| KK9 | *Hyperolius* | *kivuensis* | -2,607 | 29,757 | 1643 | 26,25 | 1 | 0 | 1 |
| KK10 | *Hyperolius* | *kivuensis* | -2,607 | 29,757 | 1643 | 28,5 | 1 | 0 | 1 |
| KK11 | *Hyperolius* | *kivuensis* | -2,607 | 29,757 | 1643 | 27,6 | 1 | 1 | 1 |
| KK12 | *Hyperolius* | *kivuensis* | -2,607 | 29,757 | 1643 | 28,2 | 1 | 0 | 2 |
| KK13 | *Hyperolius* | *kivuensis* | -2,607 | 29,757 | 1643 | 28,3 | 1 | 1 | 2 |
| KK14 | *Hyperolius* | *kivuensis* | -2,607 | 29,757 | 1643 | 25,6 | 1 | 0 | 1 |
| KK15 | *Hyperolius* | *kivuensis* | -2,607 | 29,757 | 1643 | 28,7 | 1 | 0 | 1 |
| KK16 | *Hyperolius* | *kivuensis* | -2,607 | 29,757 | 1643 | 28,65 | 1 | 0 | 2 |
| KK17 | *Hyperolius* | *kivuensis* | -2,607 | 29,757 | 1643 | 23,7 | 1 | 0 | 1 |
| KK18 | *Hyperolius* | *kivuensis* | -2,607 | 29,757 | 1643 | 28,1 | 1 | 0 | 1 |
| KK19 | *Hyperolius* | *kivuensis* | -2,607 | 29,757 | 1643 | 28,1 | 1 | 0 | 1 |
| KK20 | *Hyperolius* | *kivuensis* | -2,607 | 29,757 | 1643 | 27,15 | 1 | 0 | 2 |
| KK21 | *Hyperolius* | *kivuensis* | -2,607 | 29,757 | 1643 | 26,3 | 1 | 0 | 1 |
| K1 | *Hyperolius* | *kivuensis* | -2,607 | 29,757 | 1643 | 25,35 | 1 | 0 | 2 |
| K2 | *Hyperolius* | *kivuensis* | -1,50729 | 29,65321 | 1807 | 26,4 | 1 | 1 | 2 |
| K3 | *Hyperolius* | *kivuensis* | -1,50729 | 29,65321 | 1807 | 28 | 1 | 1 | 2 |
| K4 | *Hyperolius* | *kivuensis* | -2,607 | 29,757 | 1643 | 25,4 | 1 | 0 | 1 |
| K5 | *Hyperolius* | *kivuensis* | -1,50729 | 29,65321 | 1807 | 27,3 | 1 | 1 | 2 |
| K6 | *Hyperolius* | *kivuensis* | -1,50729 | 29,65321 | 1807 | 24,6 | 1 | 1 | 1 |
| K7 | *Hyperolius* | *kivuensis* | -2,607 | 29,757 | 1643 | 24,5 | 1 | 0 | 2 |
| K8 | *Hyperolius* | *kivuensis* | -2,607 | 29,757 | 1643 | 27,8 | 1 | 1 | 2 |
| K9 | *Hyperolius* | *kivuensis* | -1,50729 | 29,65321 | 1807 | 33,5 | 2 | 1 | 1 |
| K10 | *Hyperolius* | *kivuensis* | -1,50729 | 29,65321 | 1807 | 27,5 | 1 | 0 | 1 |
| K11 | *Hyperolius* | *kivuensis* | -1,50729 | 29,65321 | 1807 | 28,8 | 1 | 0 | 2 |
| K12 | *Hyperolius* | *kivuensis* | -1,50729 | 29,65321 | 1807 | 29 | 1 | 0 | 2 |
| K13 | *Hyperolius* | *kivuensis* | -1,50729 | 29,65321 | 1807 | 23,5 | 1 | 1 | 1 |
| K14 | *Hyperolius* | *kivuensis* | -1,50729 | 29,65321 | 1807 | 26,9 | 1 | 0 | 1 |
| K15 | *Hyperolius* | *kivuensis* | -1,50729 | 29,65321 | 1807 | 24,3 | 1 | 0 | 1 |
| K16 | *Hyperolius* | *kivuensis* | -1,50729 | 29,65321 | 1807 | 26,2 | 1 | 0 | 1 |
| K17 | *Hyperolius* | *kivuensis* | -1,50729 | 29,65321 | 1807 | 24,3 | 1 | 0 | 2 |
| K18 | *Hyperolius* | *kivuensis* | -1,50729 | 29,65321 | 1807 | 24,5 | 1 | 0 | 1 |
| K19 | *Hyperolius* | *kivuensis* | -1,50729 | 29,65321 | 1807 | 26,55 | 1 | 1 | 1 |
| K21 | *Hyperolius* | *kivuensis* | -1,50729 | 29,65321 | 1807 | 25,6 | 1 | 0 | 2 |
| K25 | *Hyperolius* | *kivuensis* | -1,50729 | 29,65321 | 1807 | 25,3 | 1 | 1 | 2 |
|  |  |  |  |  |  |  |  |  |  |
| 1Nge | *Hyperolius* | *castaneus* | -1,441667 | 29,494444 | 2879 | 25 | 1 | 1 | 1 |
| 1K | *Hyperolius* | *castaneus* | -2,485863 | 29,153058 | 1961 | 28 | 2 | 1 | 1 |
| 2K | *Hyperolius* | *castaneus* | -2,485863 | 29,153058 | 1961 | 27 | 2 | 1 | 1 |
| 2K | *Hyperolius* | *castaneus* |  |  |  |  | 2 | 1 |  |
| 3K | *Hyperolius* | *castaneus* | -2,485863 | 29,153058 | 1961 | 22,8 | 1 | 1 | 1 |
| 3K | *Hyperolius* | *castaneus* |  |  |  |  | 1 | 1 |  |
| 4K | *Hyperolius* | *castaneus* | -2,485863 | 29,153058 | 1961 | 30,5 | 2 | 1 | 1 |
| 5K | *Hyperolius* | *castaneus* | -2,485863 | 29,153058 | 1961 | 28 | 2 | 1 | 3 |
| 5K | *Hyperolius* | *castaneus* |  |  |  |  | 2 | 0 |  |
| 6K | *Hyperolius* | *castaneus* | -2,485863 | 29,153058 | 1961 | 27 | 2 | 1 | 1 |
| 6K | *Hyperolius* | *castaneus* |  |  |  |  | 2 | 1 |  |
| 7K | *Hyperolius* | *castaneus* | -2,485863 | 29,153058 | 1961 | 28 | 2 | 1 | 2 |
| 8K | *Hyperolius* | *castaneus* | -2,485863 | 29,153058 | 1961 | 26,5 | 2 | 1 | 2 |
| 9K | *Hyperolius* | *castaneus* | -2,485863 | 29,153058 | 1961 | 25 | 1 | 1 | 1 |
| 1R | *Hyperolius* | *castaneus* | -2,528683 | 29,354033 | 2379 | 22 | 1 | 1 | 1 |
| 2R | *Hyperolius* | *castaneus* | -2,528683 | 29,354033 | 2379 | 33 | 2 | 1 | 2 |
| 3R | *Hyperolius* | *castaneus* | -2,528683 | 29,354033 | 2379 | 23 | 1 | 1 | 1 |
| 4R | *Hyperolius* | *castaneus* | -2,528683 | 29,354033 | 2379 | 29 | 2 | 1 | 1 |
| 5R | *Hyperolius* | *castaneus* | -2,528683 | 29,354033 | 2379 | 31 | 2 | 1 | 1 |
| 6R | *Hyperolius* | *castaneus* | -2,528683 | 29,354033 | 2379 | 23 | 1 | 1 | 1 |
| 7R | *Hyperolius* | *castaneus* | -2,528683 | 29,354033 | 2379 | 25 | 1 | 1 | 3 |
| 8R | *Hyperolius* | *castaneus* | -2,528683 | 29,354033 | 2379 | 30 | 2 | 1 | 1 |
| 9R | *Hyperolius* | *castaneus* | -2,528683 | 29,354033 | 2379 | 22 | 1 | 1 | 1 |
| 10R | *Hyperolius* | *castaneus* | -2,528683 | 29,354033 | 2379 | 23 | 1 | 1 | 1 |
| 11R | *Hyperolius* | *castaneus* | -2,528683 | 29,354033 | 2379 | 23 | 1 | 1 | 1 |
| 12R | *Hyperolius* | *castaneus* | -2,528683 | 29,354033 | 2379 | 23 | 1 | 1 | 1 |
| 13R | *Hyperolius* | *castaneus* | -2,528683 | 29,354033 | 2379 | 30 | 2 | 1 | 1 |
| 14R | *Hyperolius* | *castaneus* | -2,528683 | 29,354033 | 2379 | 24 | 1 | 1 | 2 |
| 15R | *Hyperolius* | *castaneus* | -2,528683 | 29,354033 | 2379 | 24,5 | 1 | 1 | 2 |
| 16R | *Hyperolius* | *castaneus* | -2,528683 | 29,354033 | 2379 | 30 | 2 | 1 | 3 |
| 18R | *Hyperolius* | *castaneus* | -2,528683 | 29,354033 | 2379 | 23,5 | 1 | 1 | 2 |
| 19R | *Hyperolius* | *castaneus* | -2,528683 | 29,354033 | 2379 | 21 | 1 | 1 | 1 |
| 569 | *Hyperolius* | *castaneus* | -1,82325 | 29,36045 | 2086 | 25,38 | 1 | 1 | 1 |
| 581 | *Hyperolius* | *castaneus* | -1,82325 | 29,36045 | 2086 | 26,85 | 1 | 1 | 1 |
| 582 | *Hyperolius* | *castaneus* | -1,82325 | 29,36045 | 2086 | 24,44 | 1 | 1 | 1 |
| 583 | *Hyperolius* | *castaneus* | -1,82325 | 29,36045 | 2086 | 31,94 | 2 | 1 | 2 |
| 586 | *Hyperolius* | *castaneus* | -1,82325 | 29,36045 | 2086 | 26,13 | 1 | 1 | 2 |
| 587 | *Hyperolius* | *castaneus* | -1,82325 | 29,36045 | 2086 | 26,7 | 1 | 1 | 2 |
| 588 | *Hyperolius* | *castaneus* | -1,82325 | 29,36045 | 2086 | 27,15 | 1 | 1 | 1 |
| 589 | *Hyperolius* | *castaneus* | -1,82325 | 29,36045 | 2086 | 26,87 | 1 | 1 | 2 |
| 590 | *Hyperolius* | *castaneus* | -1,82325 | 29,36045 | 2086 | 24,82 | 1 | 1 | 1 |
| 600 | *Hyperolius* | *castaneus* | -2,48052 | 29,22847 | 2283 | 25,85 | 1 | 1 | 0 |
| 601 | *Hyperolius* | *castaneus* | -2,485863 | 29,153058 | 1961 | 18,2 | 3 | 1 | 1 |
| 602 | *Hyperolius* | *castaneus* | -2,485863 | 29,153058 | 1961 | 19,84 | 3 | 1 | 1 |
| 603 | *Hyperolius* | *castaneus* | -2,485863 | 29,153058 | 1961 | 15,53 | 3 | 1 | 0 |
| 604 | *Hyperolius* | *castaneus* | -2,485863 | 29,153058 | 1961 | 15,48 | 3 | 1 | 0 |
| 634 | *Hyperolius* | *castaneus* | -2,54601 | 29,42647 | 2190 | 25,67 | 1 | 1 | 2 |
| 640 | *Hyperolius* | *castaneus* | -2,77868 | 29,42511 | 2238 | 25,67 | 1 | 1 | 2 |
| 641 | *Hyperolius* | *castaneus* | -2,77868 | 29,42511 | 2238 | 25,95 | 1 | 1 | 1 |
| 676 | *Hyperolius* | *castaneus* | -2,485863 | 29,153058 | 1961 | 22,89 | 1 | 1 | 0 |
| 680 | *Hyperolius* | *castaneus* | -2,54601 | 29,42647 | 2190 | 25,09 | 1 | 1 | 1 |
| 681 | *Hyperolius* | *castaneus* | -2,485863 | 29,153058 | 1961 | 15,15 | 3 | 1 | 0 |
| 682 | *Hyperolius* | *castaneus* | -2,485863 | 29,153058 | 1961 | 13,92 | 3 | 1 | 0 |
| 683 | *Hyperolius* | *castaneus* | -2,485863 | 29,153058 | 1961 | 15,18 | 3 | 1 | 0 |
| 725 | *Hyperolius* | *castaneus* | -2,4478 | 29,10724 | 1813 | 22,28 | 1 | 1 | 0 |
| 728 | *Hyperolius* | *castaneus* | -2,4478 | 29,10724 | 1813 | 24,72 | 1 | 1 | 0 |
| 729 | *Hyperolius* | *castaneus* | -2,4478 | 29,10724 | 1813 | 23,16 | 1 | 1 | 0 |
| 1RR | *Hyperolius* | *castaneus* | -2,528683 | 29,354033 | 2379 | 22,7 | 1 | 1 | 1 |
| 2RR | *Hyperolius* | *castaneus* | -2,528683 | 29,354033 | 2379 | 23,7 | 1 | 1 | 3 |
| 3RR | *Hyperolius* | *castaneus* | -2,528683 | 29,354033 | 2379 | 21,3 | 1 | 1 | 2 |
| 4RR | *Hyperolius* | *castaneus* | -2,528683 | 29,354033 | 2379 | 20,6 | 1 | 1 | 1 |
| 5RR | *Hyperolius* | *castaneus* | -2,528683 | 29,354033 | 2379 | 20,3 | 1 | 1 | 1 |
| 6RR | *Hyperolius* | *castaneus* | -2,528683 | 29,354033 | 2379 | 24,1 | 2 | 1 | 1 |
| 7RR | *Hyperolius* | *castaneus* | -2,528683 | 29,354033 | 2379 | 22,2 | 1 | 1 | 1 |
| 8RR | *Hyperolius* | *castaneus* | -2,528683 | 29,354033 | 2379 | 25,2 | 1 | 1 | 2 |
| 9RR | *Hyperolius* | *castaneus* | -2,528683 | 29,354033 | 2379 | 23,3 | 1 | 1 | 1 |
| 1 | *Hyperolius* | *castaneus* | -2,528683 | 29,354033 | 2379 | 21,4 | 1 | 1 | 1 |
| 2 | *Hyperolius* | *castaneus* | -2,528683 | 29,354033 | 2379 | 20,9 | 1 | 1 | 1 |
| 17 | *Hyperolius* | *castaneus* | -2,528683 | 29,354033 | 2379 | 12 | 3 |  | 0 |
| 18 | *Hyperolius* | *castaneus* | -2,528683 | 29,354033 | 2379 | 11,6 | 3 |  | 0 |
| 19 | *Hyperolius* | *castaneus* | -2,528683 | 29,354033 | 2379 | 16,5 | 3 |  | 0 |
