# Supplementary material for: Tropical anurans mature early and die young: Evidence from eight Afromontane Hyperolius species and a meta-analysis
Source: PLoS One. 2017 Feb 9;12(2):e0171666. doi: 10.1371/journal.pone.0171666 (PMC5300166; doi:10.1371/journal.pone.0171666)
Supplement: S3 Table — This data set was used for the metaanalysis. (DOCX) [file pone.0171666.s003.docx]

**S3 Table.** Data extracted from references listed in S1 Table on age, size, gender and geographical origin of tropical anuran specimens. This data set was used for the metaanalysis.

| Species | Latitudee | Longitude | Elevation | SVL at Metamorphosis | SVL at maturity | sex | Age at maturity | Maximum SVL | Longevity |
| --- | --- | --- | --- | --- | --- | --- | --- | --- | --- |
|  | [°] | [°] | [m] | [mm] | [mm] | 1=male, 2=female] | [n LAGs] | [mm] | [n LAGs] |
| *Amietophrynus pentoni* | 18,45 | -15,7 | 8 |  | 54 | 1 | 2 | 63,5 | 6 |
| *Amietophrynus pentoni* | 18,45 | -15,7 | 8 |  | 58 | 2 | 2 | 71 | 6 |
| *Atelopus chiriquiensis* | 9,18 | -82,8 | 2200 |  | 37,3 | 2 | 2 | 46,1 | 4 |
| *Atelopus peruensis* | 5,5 | -79,9 | 3500 |  | 33,6 | 1 | 3 | 45,5 | 6 |
| *Atelopus peruensis* | 5,5 | -79,9 | 3500 |  | 42,1 | 2 | 3 | 44,71 | 6 |
| *Atelopus spec.* | 9,3 | -79,2 | 600 |  | 25,1 | 1 | 2 | 30,3 | 3 |
| *Boophis occidentalis* | -14,3 | 47,9 | 170 | 11,4 | 50 | 1 | 4 | 60 | 9 |
| *Boophis williamsi* | -19,35 | 47,25 | 2285 |  | 37,1 | 1 | 1 | 40 | 6 |
| *Boophis williamsi* | -19,35 | 47,25 | 2285 |  | 39 | 2 | 2 | 42,6 | 5 |
| *Bufo bankorensis* | 22,05 | 120,6 | 230 | 12,1 | 62,5 | 1 | 1 | 82,7 | 5 |
| *Cophixalus ornatus* | -19 | 146,02 | 980 |  | 16 | 1 | 4 | 25 | 14 |
| *Duttaphrynus melanostictus* | 20,3 | 85,86 | 56 | 10,5 | 41 | 1 | 1 | 78 | 5 |
| *Duttaphrynus melanostictus* | 20,3 | 85,86 | 56 | 10,5 | 41 | 2 | 1 | 103 | 11 |
| *Dyscophus antongilii* | -16,85 | 49,2 | 486 |  | 52,1 | 1 | 3 | 64,2 | 7 |
| *Dyscophus antongilii* | -16,85 | 49,2 | 486 |  | 66,5 | 2 | 3 | 98,9 | 11 |
| *Dyscophus guineti* | -18,7 | 48,6 | 680 |  | 61,3 | 1 | 2 | 78,1 | 6 |
| *Dyscophus guineti* | -18,7 | 48,6 | 680 |  | 66,8 | 2 | 2 | 112,4 | 7 |
| *Euphlyctis cyanophlyctis* | 21,68 | 87,3 | 14 | 25 | 41 | 1 | 1 | 52 | 5 |
| *Euphlyctis cyanophlyctis* | 21,68 | 87,3 | 14 | 25 | 40 | 2 | 1 | 60 | 5 |
| *Euphlyctis cyanophlyctis* | 15,3 | 75,5 | 626 | 25 | 49 | 2 | 2 | 88 | 7 |
| *Euphlyctis cyanophlyctis* | 15,3 | 75,5 | 626 | 25 | 34 | 1 | 1 | 60 | 5 |
| *Euphlyctis hexadactylus* | 21,68 | 87,3 | 14 | 12 | 57 | 1 | 1 | 140 | 13 |
| *Euphlyctis hexadactylus* | 21,68 | 87,3 | 14 | 12 | 75 | 2 | 1 | 145 | 5 |
| *Fejervarya cancrivora* | -6,7 | 106,5 | 220 | 14,5 |  | 1 | 0 | 56,7 | 1 |
| *Fejervarya limocharis* | 15,3 | 75,5 | 626 | 15,7 | 22 | 1 | 0 | 35 | 3 |
| *Hoplobatrachus tigerinus* | 14,8 | 78,2 | 11 |  | 42 | 1 | 0,85 | 140 | 7 |
| *Hoplobatrachus tigerinus* | 14,8 | 78,2 | 11 |  | 40 | 2 | 0,85 | 135 | 7 |
| *Hylarana nigrovittata* | 13,4 | 101,8 | 110 | 16,1 | 37,2 | 1 | 0 | 67,9 | 9 |
| *Hylarana nigrovittata* | 13,4 | 101,8 | 110 | 16,1 | 33,2 | 2 | 1 | 53,9 | 6 |
| *Hyperolius castaneus* | -2,4 | 29,1 | 2100 | 11,5 | 20,3 | 1 | 0 | 27,2 | 3 |
| *Hyperolius castaneus* | -2,4 | 29,1 | 2100 | 11,5 | 24,1 | 2 | 1 | 33 | 3 |
| *Hyperolius cinnamomeoventris* | -2,6 | 29,8 | 1643 | 8,3 | 18,6 | 1 | 1 | 22,5 | 3 |
| *Hyperolius discodactylus* | -2,5 | 29,4 | 2389 | 11 | 29,4 | 1 | 1 | 34,4 | 2 |
| *Hyperolius glandicolor* | -1,6 | 29,4 | 2287 |  | 20,5 | 1 | 0 | 27 | 1 |
| *Hyperolius glandicolor* | -1,6 | 29,4 | 2287 |  | 26 | 2 | 1 | 34,9 | 2 |
| *Hyperolius kivuensis* | -2,6 | 29,8 | 1643 | 12,2 | 25,4 | 1 | 1 | 33,9 | 3 |
| *Hyperolius lateralis* | -2,6 | 29,8 | 1643 | 11,5 | 19 | 1 | 0 | 23,9 | 1 |
| *Hyperolius rwandae* | -2,6 | 29,8 | 1643 | 9 | 18,2 | 1 | 1 | 20,2 | 4 |
| *Hyperolius viridiflavus* | -2,6 | 29,8 | 1643 | 14 | 25,2 | 1 | 1 | 33,7 | 2 |
| *Hypsiboas rosenbergi* | 9,09 | 79,7 | 87 | 20,7 |  | 1 | 1 | 89 |  |
| *Hypsiboas rosenbergi* | 9,09 | 79,7 | 87 | 20,7 |  | 2 | 1 | 89 |  |
| *Leptodactylus fallax* | 16,75 | -61,16 | 100 |  | 128 | 1 | 3 | 190 | 6 |
| *Leptodactylus fallax* | 16,75 | -61,16 | 100 |  | 130 | 2 | 3 | 280 | 7 |
| *Limnonectes macrodon* | -6,7 | 106,5 | 220 |  | 62 | 1 | 1 | 105,2 | 5 |
| *Mantella baroni* | -20,8 | 47,3 | 1400 |  | 18,6 | 1 | 1 | 28,5 | 3 |
| *Mantella baroni* | -20,8 | 47,3 | 1400 |  | 26,9 | 2 | 1 | 30,1 | 2 |
| *Mantella bernhardi* | -21,4 | 47,5 | 500 |  | 17,1 | 1 | 1 | 21,8 | 3 |
| *Mantella bernhardi* | -21,4 | 47,5 | 500 |  | 18,1 | 2 | 1 | 20,1 | 3 |
| *Mantella cowani* | -20,8 | 47,3 | 1400 |  | 15,1 | 1 | 1 | 28,9 | 2 |
| *Mantella cowani* | -20,8 | 47,3 | 1400 |  | 18,1 | 2 | 1 | 31,4 | 3 |
| *Mantella crocea* | -18,35 | 48,4 | 910 |  | 14,8 | 1 | 1 | 19,5 | 4 |
| *Mantella expectata* | -22,16 | 45,16 | 850 | 11,1 | 20,2 | 1 | 1 | 23,98 | 3 |
| *Mantella expectata* | -22,16 | 45,16 | 850 | 11,1 | 24,2 | 2 | 1 | 27,6 | 3 |
| *Mantella laevigata* | -15,3 | 50,05 | 615 |  | 23,7 | 1 | 2 | 24,7 | 2 |
| *Mantella laevigata* | -15,3 | 50,05 | 615 |  | 24,6 | 2 | 1 | 27 | 2 |
| *Mantella nigricans* | -15,3 | 50,05 | 615 |  | 24,1 | 1 | 1 | 26 | 4 |
| *Mantella nigricans* | -15,3 | 50,05 | 615 |  | 26,5 | 2 | 1 | 28,3 | 3 |
| *Mantella pulchra* | -18,35 | 48,4 | 910 |  | 18,4 | 1 | 1 | 22,7 | 3 |
| *Mantella pulchra* | -18,35 | 48,4 | 910 |  | 21,3 | 2 | 1 | 28,2 | 4 |
| *Mantidactylus pauliani* | -19,35 | 47,25 | 2285 |  | 25 | 1 | 2 | 35,8 | 8 |
| *Mantidactylus pauliani* | -19,35 | 47,25 | 2285 |  | 27 | 2 | 2 | 35,3 | 8 |
| *Microhyla ornata* | 15,3 | 75,5 | 626 |  | 15 | 1 | 1 | 26,5 | 4 |
| *Microhyla ornata* | 15,3 | 75,5 | 626 |  | 13 | 2 | 1 | 28 | 4 |
| *Micryletta steinegeri* | 22,05 | 120,6 | 230 |  | 22,6 | 1 | 0 | 29,2 | 4 |
| *Micryletta steinegeri* | 22,05 | 120,85 | 345 |  | 22,58 | 1 | 0 | 29,15 | 4 |
| *Nimbaphrynoides occidentalis* | 7,64 | -8,35 | 1200 | 6 | 10 | 1 | 0,3 | 22 | 2 |
| *Nimbaphrynoides occidentalis* | 7,64 | -8,35 | 1200 | 6 | 12 | 2 | 0,3 | 27 | 5 |
| *Phrynobatrachus guineensis* | 5,83 | -7,33 | 200 |  |  | 1 | 0,3 |  | 1 |
| *Phrynobatrachus guineensis* | 5,83 | -7,33 | 200 |  |  | 2 | 0,3 |  | 1 |
| *Polypedates maculatus* | 21,68 | 87,3 | 14 | 30 | 35 | 1 | 1 | 70 | 4 |
| *Polypedates maculatus* | 21,68 | 87,3 | 14 | 31 | 41 | 2 | 1 | 83 | 6 |
| *Polypedates teraiensis* | 21,68 | 87,3 | 14 |  | 45 | 1 | 1 |  |  |
| *Polypedates teraiensis* | 21,68 | 87,3 | 14 |  | 41 | 2 | 1 | 78 | 4 |
| *Scaphiophryne gottlebei* | -22,16 | 45,16 | 850 | 17 | 25,8 | 1 | 1 | 31,7 | 2 |
| *Scaphiophryne gottlebei* | -22,16 | 45,16 | 850 | 17 | 32,1 | 2 | 1 | 36,1 | 2 |
