# Supplementary material for: Tropical anurans mature early and die young: Evidence from eight Afromontane Hyperolius species and a meta-analysis
Source: PLoS One. 2017 Feb 9;12(2):e0171666. doi: 10.1371/journal.pone.0171666 (PMC5300166; doi:10.1371/journal.pone.0171666)
Supplement: S4 Table — (DOCX) [file pone.0171666.s004.docx]

**S4 Table**. References used for the meta-analysis of demographic life-history traits, but not listed in the main text.

1. Andreone F, Crottini A, Rabemananjara F, Randrianirina JE, Razafindrabe T, Tessa G. Age structure, population estimate and Bd-status of two Critically Endangered frogs from the Ankaratra Massif (Madagascar), *Boophis willliamsi* and *Mantidactylus pauliani* (Amphibia: Mantellidae). In: Capula M, Corti C, editors. Scripta Herpetologica Studies on Amphibians and Reptiles in honour of Benedetto Lanza 2014. p. 17-29.
2. Nayak S, Mahapatra PK, Mishra G, Dutta HM. Age determination by skeletochronology in the Common Indian Toad *Bufo melanostictus* SCHNEIDER, 1799 (Anura: Bufonidae). Herpetozoa. 2007; 19: 111-9.
3. Tessa G, Guarino FM, Randrianirina JE, Andreone F. Age structure in the false tomato frog *Dyscophus guineti* from eastern Madagascar compared to the closely related *D. antongilii* (Anura, Microhylidae). Afr J Herpetol. 2011; 60: 84-8. doi: 10.1080/21564574.2011.561881. PMID: WOS:000293176100008.
4. Kulkarni JT, Pancharatna K. Age related changes in ovarian follicular kinetics in the Indian skipper frog *Rana cyanophlyctis* (Schn). J Biosci. 1996; 21: 699-710. doi: 10.1007/bf02703146. PMID: WOS:A1996VU57500009.
5. Pancharatna K, Chandran S, Kumbar S. Phalangeal growth marks related to testis development in the frog *Rana cyanophlyctis*. Amphibia-Reptilia. 2000; 21: 371-379.
6. Gramapurohit NP, Shanbhag BA, Saidapur SK. Post-metamorphic growth, sexual maturation and body size dimorphism in the skipper frog, *Euphlyctis cyanophlyctis* (Schneider). Herpetol J. 2005; 15: 113-119.
7. Pancharatna K, Deshpande SA. Skeletochronological data on age, body size and mass in the Indian Cricket Frog *Limnonectes limnocharis* (BOIE, 1835) (Anura: Ranidae). Herpetozoa. 2003; 16: 41-50.
8. Guarino FM, Tessa G, Mercurio V, Andreone F. Rapid sexual maturity and short life span in the blue-legged frog and the rainbow frog from the arid Isalo Massif, southern-central Madagascar. Zoology. 2010; 113:378-384.
9. Kumbar SM, Pancharatna K. Determination of age, longevity and age at reproduction of the frog *Microhyla ornata* by skeletochronology. J Biosci. 2001; 26: 265-270. doi: 10.1007/bf02703650. PMID: WOS:000169454400019.
10. Mahapatra PK, Nayak S, Dutta SK. Age estimates for a population of the Indian Tree Frog *Polypedates maculatus* (GRAY, 1833) (Anura: Rhacophoridae). Herpetozoa. 2008; 21: 31-40.
11. Andia BN, Dixit PK, Behera S, Mishra G, Behera HN. Assessment of age and growth in common tree frog, *Polypedates maculatus* Gray by skeletochronology. Proc Nat Acad Sci India. 2010; 80(B): 221-229. PMID: ISI:000282831600004.
12. Felton A, Alford RA, Felton AM, Schwarzkopf L. Multiple mate choice criteria and the importance of age for male mating success in the microhylid frog, *Cophixalus ornatus*. Behavioral Ecology and Sociobiology. 2006; 59(6):786-95. doi: 10.1007/s00265-005-0124-6. PMID: WOS:000236199600009.
13. Kluge AG. The life history, social organization, and parental behavior of *Hyla rosenbergi* Boulenger, a nest-building gladiator frog. Miscellaneous publications, Museum of Zoology, University of Michigan. 1981; 160:1-170.
14. Castanet J, Pinto S, Loth MM, Lamotte M. Age individuel, longevite et dynamique de croissance osseuse chez un amphibien vivipare, *Nectophrynoides occidentalis* (Anoure, Bufonide). Annales Des Sciences Naturelles, Zoologie et Biologie Animale. 2000; 21(1):11-7.
15. Rödel M, Rudolf V, Frohschammer S, Linsenmair K. Life history of a West-African tree-hole breeding frog, *Phrynobatrachus guineensis* Guibé and Lamotte, 1961 (Amphibia: Anura: Petropedetidae). In: Lehtinen RM, editor. Ecology and evolution of phytotelm-breeding anurans: Misc. Publ. Mus. Zool. Univ. Mich.; 2004. p. 31-44.
16. Dutta SK, Nayak S, Mahapatra C, Das M, Mahpatra PK. Skeletochronology of Anura (Amphibia). Proceedings of the National Academy of Sciences India. 2011; 81 (B):83-102. PMID: ISI:000289536800005.
17. Pancharatna K, Kumbar SM. Estimation of age and longevity of the Indian Bullfrog *Hoplobatrachus tigerinus* (DAUDIN, 1802): A skeletochronological study. Herpetozoa. 2005; 18(3/4):147-153.
18. Gramapurohit NP, Shanbhag BA, Saidapur SK. Growth, sexual maturation and body size dimorphism in the Indian bullfrog, *Hoplobatrachus tigerinus* (Daud.). Herpetologica. 2004; 60(4):414-419.
19. Andia BN, Behera HN. Life span, sexual maturity and age determination of Indian Bull Frog, *Rana tigrina* using skeletochronology. Proceedings of the National Academy of Sciences India. 2005; 75 (B)(Part 4):261-70. PMID: BCI:BCI200600242048.
